# Changing Course: Semaphorins and Plexins as Novel Therapeutic Opportunities and Key Regulators of Pancreatic Cancer Microenvironment

**DOI:** 10.3390/ijms27219788

**Published:** 2026-10-31

**Authors:** Nicolò Bertalmio, Francesca Anna Lourdes Marino, Serena Brundu, Serena Rustichelli, Enrico Giraudo

**Affiliations:** 1 Tumor Microenvironment Laboratory, INOC—Istituto Nazionale Oncologico Candiolo, IRCCS, 10060 Candiolo, Turin, Italy; nicolo.bertalmio@inoc.it (N.B.); francesca.marino@inoc.it (F.A.L.M.); serena.brundu@inoc.it (S.B.); serena.rustichelli@inoc.it (S.R.); 2 Department of Science and Drug Technology, University of Turin, 10125 Turin, Italy

**Keywords:** pancreatic ductal adenocarcinoma, tumor microenvironment, Semaphorins and Plexins

## Abstract

Pancreatic ductal adenocarcinoma (PDAC) remains one of the deadliest solid tumors, and despite recent advances in surgical and systemic therapies, outcomes remain poor, rooted as much in the genetic mutations that initiate the disease as in a fibrotic, poorly vascularized microenvironment marked by scarce immune infiltration and a dense network of nerve fibers. Semaphorins, together with their receptors plexins and co-receptors neuropilins, have emerged as pivotal orchestrators of this microenvironment, shaping crosstalk between malignant cells, fibroblasts, macrophages, the endothelium, and peripheral neurons. This review examines how individual semaphorin family members drive, or in select contexts restrain, stromal activation, immune exclusion, pathological angiogenesis and neural remodeling. Building on this mechanistic framework, we explore the clinical relevance that semaphorin-mediated signaling holds for PDAC, from its potential as a prognostic and predictive biomarker to its growing promise as a therapeutic target, positioning this axis as a compelling frontier for improving outcomes in a disease that has long resisted meaningful therapeutic advances.

## 1. PDAC Clinical Burden: How Microenvironmental Complexity Worsens Prognosis

Pancreatic ductal adenocarcinoma (PDAC) is the predominant histological subtype of pancreatic cancer and remains one of the most lethal solid malignancies [1,2]. Its clinical outcome is largely driven by a combination of late diagnosis [3], early metastatic dissemination [4], and profound resistance to systemic therapies [5]. Despite advances in surgical management and the introduction of increasingly effective multi-agent chemotherapy regimens [6,7], only a minority of patients present with resectable disease amenable to curative-intent surgery, and long-term survival remains poor [7,8]. Recent therapeutic developments, including molecularly targeted approaches [9], have expanded the treatment landscape. Notably, daraxonrasib, a first-in-class RAS(ON) multi-selective inhibitor, improved survival in previously treated metastatic pancreatic cancer [10,11]. However, its role is currently limited to the second-line setting, and acquired resistance [12] reflects the broader biological complexity and adaptability of PDAC that continues to constrain the clinical impact of targeted therapies [13]. The aggressive behavior of PDAC cannot be explained solely by the genetic alterations intrinsic to malignant epithelial cells [14]. Rather, tumor progression occurs within a highly organized and dynamic tumor microenvironment (TME) that supports tumor growth, invasion, immune escape, therapy resistance, and neural remodeling. The TME consists of a complex network of malignant cells, stromal populations, immune cells, extracellular matrix (ECM) components, vascular structures, and neural elements that engage in extensive bidirectional crosstalk [15,16]. Recent advances in single-cell RNA sequencing and spatial transcriptomics have significantly refined our understanding of the PDAC microenvironment, revealing striking cellular and spatial heterogeneity. These approaches have identified distinct functional states of cancer-associated fibroblasts (CAFs), macrophages, and endothelial and immune cells, demonstrating that tumor progression is driven by dynamic cell-state transitions and extensive ligand-receptor communication rather than by individual cell populations alone [17,18]. These observations support a broader view of PDAC as a disease sustained by reciprocal communication between malignant cells and their surrounding microenvironment. Among the signaling systems involved in these interactions, pathways originally identified as regulators of axonal development have attracted increasing attention. Semaphorins (SEMAs) and their receptors, plexins (PLXNs) and neuropilins (NRPs), regulate cell migration, adhesion, cytoskeletal organization, and tissue patterning, and have subsequently been implicated in cancer biology [19]. Their effects in PDAC appear to be particularly context-dependent, with individual family members capable of promoting or restraining tumor progression depending on their cellular source, receptor repertoire, and downstream signaling.

## 2. Understanding Semaphorin-Mediated Signaling in the Regulation of PDAC Tumor Biology

**Figure 1:**
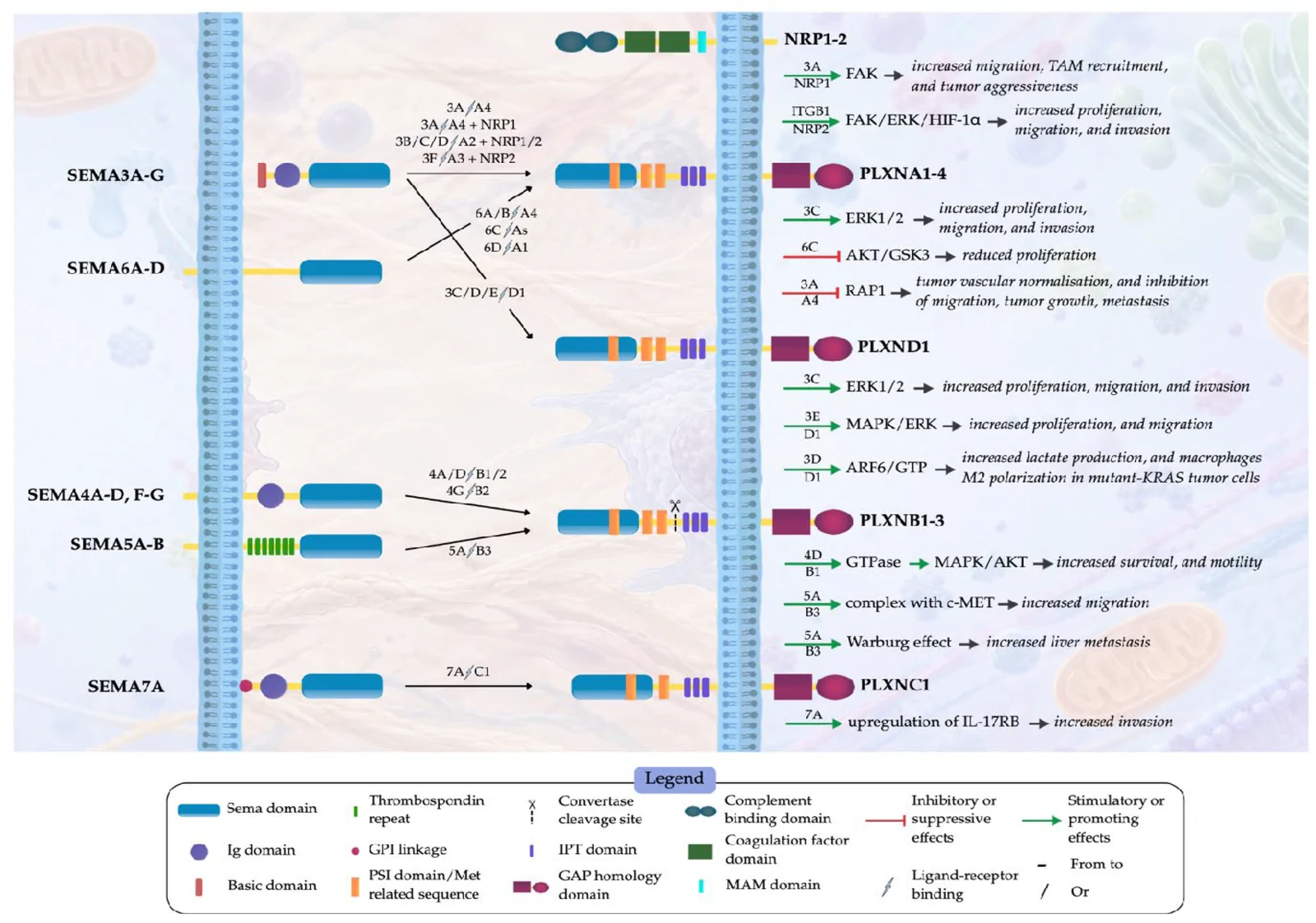
Semaphorin-plexin interactions and downstream signaling in PDAC. Schematic representation of the major semaphorins (SEMAs) and their corresponding plexin (PLXN) and neuropilin (NRP) receptors, highlighting their interactions and downstream signaling pathways in PDAC. Green arrows denote stimulatory or promoting effects, whereas red arrows indicate inhibitory or suppressive effects. Abbreviations: FAK: focal adhesion kinase; ITGB1: integrinβ1; ERK: extracellular signal-regulated kinase; HIF-1α: hypoxia-inducible factor-1α; PI3K: phosphoinositide 3-kinase; AKT: protein kinase B; GSK3: glycogen synthase kinase-3; RAP1: Ras-related protein 1; MAPK: mitogen-activated protein kinase; ARF6: ADP-ribosylation factor 6; GTP: guanosine triphosphate; GTPase: guanosine triphosphatase; c-MET: hepatocyte growth factor receptor; IL-17RB: interleukin-17 receptor B; GPI: glycophosphatidylinositol; PSI: plexin-semaphorin-integrin; IPT: Ig-like, plexins, transcription factors; GAP: GTPase-activating protein; MAM: meprin, A-5 protein, and receptor protein-tyrosine phosphatase mu. Created using the free version of Canva (https://www.canva.com).

SEMAs constitute a large family of secreted and membrane-associated guidance molecules that, through their interaction with PLXNs, NRPs, and additional co-receptors, regulate a broad spectrum of cellular processes extending well beyond their canonical roles in neuronal and vascular development [19]. Mounting evidence indicates that dysregulation of SEMA signaling contributes to cancer progression by modulating tumor cell proliferation, survival, adhesion, migration, and invasion, as well as angiogenesis, immune responses, and therapy resistance. Importantly, SEMAs and their receptors can exert either tumor-promoting or tumor-suppressive effects, depending on the specific ligand-receptor complex, cellular context and downstream signaling partners [20] (Figure 1). In particular, class 3 semaphorins are mainly secreted proteins whose signaling is mediated by NRPs in association with PLXNs, whereas other SEMA classes can interact directly with PLXNs or form receptor complexes with integrins and receptor tyrosine kinases (RTKs). Engagement of these complexes can regulate cytoskeletal dynamics and cell adhesion through small GTPases (e.g., RhoA/Rac1/Cdc42) [21], while also intersecting with canonical oncogenic pathways such as phosphoinositide 3-kinase/protein kinase B (PI3K/AKT) [22] and mitogen-activated protein kinase/extracellular signal-regulated kinase (MAPK/ERK) [23]. These signaling mechanisms appear particularly relevant in PDAC, a tumor characterized by.

Extensive stromal remodeling, profound immune suppression, and marked cellular plasticity. Several SEMAs, including SEMA3A [24,25], SEMA3C [26], SEMA3D [27], SEMA3E [28], and SEMA4D [29] have been implicated in PDAC biology, although their functional effects are not uniform. SEMA signaling can act directly on malignant epithelial cells to promote migratory and invasive phenotypes [30,31], while simultaneously affecting endothelial cells [24], fibroblasts [32], and immune populations within the TME [33]. Thus, SEMAs and their receptors also function as key mediators of TME crosstalk rather than strictly cell-autonomous signals.

For instance, NRP1-dependent SEMA3A signaling, can promote focal adhesion kinase (FAK)-dependent signaling, favoring cytoskeletal remodeling, migration, and resistance to anoikis, and activating the PI3K/Akt pathway in basal-like PDAC cells [25]. While NRP1-dependent SEMA3A signaling has been linked to protumor phenotypes, engagement of PlexinA4 by an NRP1-independent SEMA3A variant was shown to exert an antitumor effect in PDAC models, promoting tumor vascular normalization and reducing tumor growth and metastatic dissemination [24]. Moreover, overexpression of SEMA3A via an AAV-8 gene delivery system hampered tumor growth and liver metastasis formation, and reduced tumor hypoxia by inducing vessel normalization in a transgenic mouse model of neuroendocrine pancreatic cancer Rip1-Tag2 [34,35] and in a syngeneic orthotopic mouse model of PDAC [24]. These findings further underscore the receptor-dependent nature of SEMA3A signaling and suggest that the balance between NRP1- and PlexinA4-mediated pathways may critically influence whether SEMA3A promotes or restrains PDAC progression. Beyond this receptor balance, SEMA3A activity may also be shaped by growth factors that compete for NRP1. Vascular endothelial growth factor (VEGF)-A165 and class 3 SEMAs bind overlapping sites on the receptor and partially compete for occupancy [36]. In the hypoxic PDAC microenvironment, high VEGF-A may therefore limit SEMA3A binding to NRP1 and shift signaling from repulsive, anti-angiogenic cues [37] toward pro-survival and pro-angiogenic responses. Because PLXNs relay the SEMA signal by inactivating Rap and R-Ras, thereby reducing integrin-mediated adhesion [38,39], reduced SEMA3A engagement of NRP1 may also weaken this anti-adhesive, anti-migratory input.

As another secreted class 3 SEMA, SEMA3C can engage NRP1/2 and PLXN receptors, among them PLXNA family members and PLXND1 [40]. In PDAC, SEMA3C has been associated with tumor-promoting activity, with activation of the ERK1/2 pathway contributing to increased proliferation, migration, and invasion [41]. This axis is particularly relevant because it connects semaphorin signaling with the MAPK pathway, a central oncogenic signaling network in PDAC. Accordingly, inhibition of SEMA3C has been proposed as a strategy to increase the sensitivity of KRAS- or MEK-dependent pancreatic cancer cells to MAPK pathway inhibition [41,42]. SEMA3D represents another secreted semaphorin with a predominantly tumor-promoting role in PDAC. SEMA3D signaling involves NRPs and PLXND1, and its expression is increased during pancreatic tumor progression [43]. Mechanistically, SEMA3D has been linked to the acquisition of invasive and metastatic properties through an autocrine interaction between pancreatic cancer cells and, potentially, paracrine communication with neural cells [43]. Genetic depletion of SEMA3D reduces invasive and metastatic behavior in experimental models, supporting the concept that the SEMA3D-PLXND1 axis contributes directly to PDAC dissemination [44]. In contrast to most class 3 SEMAs, SEMA3E can bind directly to PLXND1 without requiring NRPs, providing a relatively distinct signaling configuration [45]. In pancreatic cancer, SEMA3E has been associated with tumor progression and poor outcome, while experimental studies indicate that its overexpression promotes proliferation and migration through activation of the MAPK/ERK pathway [28]. This observation highlights how different SEMAs can converge on common oncogenic pathways despite engaging distinct receptor complexes.

**Figure 2:**
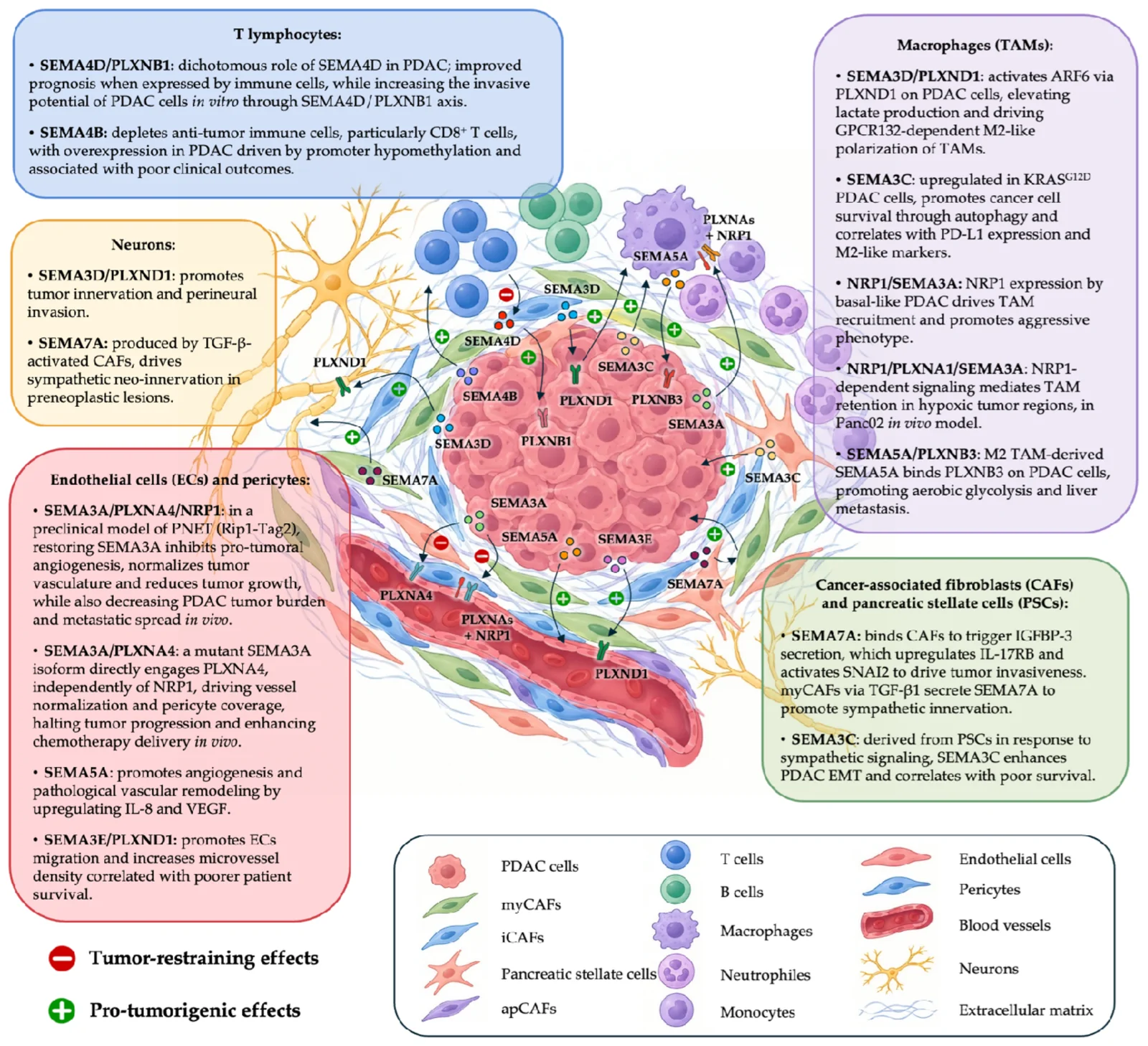
Semaphorin signaling networks shape the PDAC tumor microenvironment. SEMAs and their receptors, PLXNs and NRPs, coordinate crosstalk between PDAC tumor cells and the surrounding stroma, including cancer-associated fibroblasts, pancreatic stellate cells, tumor-associated macrophages, tumor-associated neutrophils, T lymphocytes, endothelial cells, pericytes and peripheral neurons. Depending on the ligand–receptor pair and cellular context, these interactions can either promote (+) or restrain (−) cancer progression by influencing stromal activation, immune exclusion, pathological angiogenesis, and tumor innervation. Abbreviations: ARF6: ADP-ribosylation factor 6; EMT: epithelial–mesenchymal transition; GPCR132: G protein-coupled receptor 132; IGFBP-3: insulin-like growth factor-binding protein 3; IL-8: interleukin-8; IL-17RB: interleukin-17 receptor B; KRAS: Kirsten rat sarcoma viral oncogene homolog; NRPs: neuropilins; PD-L1: programmed death-ligand 1; PLXNs: plexins; PNET: pancreatic neuroendocrine tumor; SEMAs: semaphorins; SNAI2: Snail family transcriptional repressor 2; TGF-β: transforming growth factor-beta; VEGF: vascular endothelial growth factor. “Created in BioRender. Bertalmio, N. (2026) https://BioRender.com/vre9o84; with elements from NIH BioArt Source (https://bioart.niaid.nih.gov/).

Among membrane-associated SEMAs, SEMA4D signals predominantly through PLXNB1. In PDAC, engagement of PLXNB1 activates small GTPase signaling, including RhoA, and promotes phosphorylation of MAPK and AKT, thereby enhancing tumor cell motility and invasive capacity [46]. Importantly, SEMA4D signaling also has broader effects on the TME and immune compartment, consistent with the ability of SEMA-PLXN pathways to regulate cell migration and immune cell organization [46]. Consequently, SEMA4D may represent a link between tumor-intrinsic invasive signaling and microenvironmental remodeling. SEMA5A is another membrane-associated semaphorin with tumor-promoting activity. Its interaction with PLXNB3 stimulates c-MET signaling and enhances pancreatic cell migration [47]. In experimental models, increased SEMA5A expression is associated with tumor growth and metastatic dissemination, while the SEMA5A-PLXNB3 axis has also been implicated in angiogenic responses [48]. This pathway illustrates the capacity of PLXNs to function as signaling platforms that integrate SEMA cues with receptor tyrosine kinase pathways, thereby coupling extracellular guidance signals to established pro-tumorigenic signaling networks. In contrast to the tumor-promoting effects described above, SEMA6C has been proposed to exert tumor-suppressive activity in PDAC. SEMA6C signaling inhibits the AKT/GSK3 axis, resulting in reduced cyclin D1 expression and suppression of cell proliferation. Loss or downregulation of SEMA6C may therefore favor cell-cycle progression, and this observation has prompted interest in the potential therapeutic relevance of combining restoration/targeting of this pathway with CDK4/6 inhibition [49]. The complexity of SEMA signaling stems from extensive crosstalk with other cell-surface systems. By associating with integrins and RTKs, PLXNs and NRPs integrate extracellular cues to control actin dynamics, adhesion, survival, and proliferation. Consequently, the biological response to a given SEMA ligand depends not on its absolute expression level, but on the cellular receptor repertoire and the local TME [19]. In PDAC, this receptor-dependent plasticity is particularly critical given the highly heterogeneous tumor architecture and its dense, immunosuppressive desmoplastic stroma [19]. Overall, SEMA-NRP-PLXN signaling represents a multifaceted axis linking tumor-intrinsic pathways to microenvironment remodeling (Figure 2), with major roles in invasion, metastasis, angiogenesis, immune evasion, and drug resistance. Harnessing this pathway for biomarker-guided or targeted therapies will require a more precise mapping of ligand-receptor specificity across distinct cell types within the tumor niche.

## 3. Microenvironmental Remodeling Through SEMA/PLXN-Driven Cellular Crosstalk

### 3.1. Pancreatic Stellate Cells and Cancer-Associated Fibroblasts

Pancreatic stellate cells (PSCs) in PDAC respond to tumor-derived and stromal signals, particularly transforming growth factor β (TGF-β) and platelet-derived growth factor (PDGF) [50], by activating into CAFs, which drive aggressive disease progression and poor outcomes through functional specialization [51]. Multi-omics profiling resolved this heterogeneity into three major distinct CAF subpopulations [52] shaped by local tumor and stromal cues: inflammatory CAFs (iCAFs) suppress immunity by secreting immunomodulatory molecules, including interleukin-6 (IL-6), to modulate immune cell recruitment and function [53,54]; myofibroblastic CAFs (myCAFs) remodel the extracellular matrix through processes including collagen production, fueling the extensive desmoplastic reaction characteristic of PDAC [54,55]; and antigen-presenting CAFs (apCAFs) express MHC class II molecules but lack classical costimulatory molecules, a profile that limits their immunostimulatory capacity [56], though their precise immunological role remains to be fully elucidated. Despite this functional diversity, CAF states are not fixed lineages but rather maintain functional plasticity [57] and are dynamically regulated by tumor microenvironmental cues, including SEMA-PLXN signaling, which orchestrates CAF–tumor cell interactions and modulates both CAF and tumor cell behavior in PDAC. A key example of this tumor–CAF crosstalk occurs at the invasive margin, where tumor cells leverage ATPase Na^+^/K^+^ Transporting Subunit Alpha 1 (ATP1A1)-mediated trafficking to enhance plasma membrane expression of SEMA7A, which engages the cognate receptor PLXNC1 on CAFs to promote their activation toward an invasion-supporting phenotype. Through SEMA7A-mediated signaling, CAFs are reprogrammed to secrete elevated levels of insulin-like growth factor-binding protein-3 (IGFBP-3), which acts as a paracrine signal that upregulates interleukin-17 receptor B (IL-17RB) expression on invading tumor cells, amplifying interleukin-17B (IL-17B) signaling and, ultimately, enhancing Snail Family Member 2 (SNAI2)-mediated epithelial–mesenchymal plasticity. Through this SEMA7A/CAF/IGFBP-3 axis, tumor cells at the invasive margin hijack CAF secretory functions to establish a feed-forward cascade of invasion-promoting signals at the PDAC leading edge, exemplifying how tumor-derived cues dynamically shape CAF activation during metastatic progression [58]. Interestingly, SEMA7A is also a neurotrophic ligand, as TGF-β1 was found to stimulate the formation of myCAFs from PSCs and their release of SEMA7A, thereby promoting neurite outgrowth and neural remodeling [59]. PSCs also promote PDAC progression through sympathetic nerve signaling-driven SEMA3C upregulation, which enhances EMT. Clinically, high intratumoral SEMA3C expression, along with elevated NRP1/NRP2 receptors, strongly associates with poor survival in PDAC patients. Mechanistically, SEMA3C functions through autocrine signaling to amplify PSC proliferation and simultaneously enhances tumor cell invasion while increasing SNAI2 and vimentin expression in both murine and human PDAC cells. Beyond direct effects on tumor cells, SEMA3C treatment reprograms PSCs toward an iCAF-like state, establishing a feed-forward loop in which SEMA3C coordinates changes in both tumor cells and PSCs to promote PDAC aggressiveness through sympathetic nerve-derived signaling [60]. Collectively, these findings establish semaphorins as critical regulators of CAF function and plasticity throughout PDAC progression, operating simultaneously at primary and metastatic sites to orchestrate invasion, immune evasion, and stromal support, thereby positioning SEMA-NRP-PLXN axes as compelling therapeutic targets to disrupt the tumor–stromal partnership that drives PDAC aggressiveness.

### 3.2. Immune Microenvironment

PDAC is generally an immunologically “cold” tumor, with low immunogenicity, sparse immune infiltration, and a predominantly immunosuppressive TME [61,62]. Yet infiltration varies considerably between patients, and composition carries prognostic value: myeloid-rich tumors are associated with poorer survival, whereas lymphocyte-rich tumors correlate with better overall survival [63]. Immune composition also shifts with progression, as advanced stages show increased regulatory T cells, myeloid-derived suppressor cells [64,65], and other immunoregulatory populations that limit immunotherapy efficacy [66]. Neoadjuvant therapy further reshapes T cell infiltration and the effector-suppressor balance, though effects vary across regimens [67,68]. Identifying the molecular cues that govern immune cell recruitment, polarization, and function in PDAC is therefore essential, and semaphorins and their receptors are promising candidates [33].

The myeloid compartment predominates the immune microenvironment of PDAC, with tumor-associated macrophages (TAMs) representing the most abundant immune cell population in the PDAC microenvironment, recruited by tumor- and stroma-derived signals, including IL-6, which direct circulating monocyte infiltration and differentiation into TAMs within the stromal microenvironment [69,70,71]. Once established, TAMs are predominantly skewed toward an immunosuppressive M2-like polarization state (CD163^+^, CD204^+^) characterized by secretion of interleukin-10 (IL-10) and TGF-β, which collectively suppress CD8^+^ T cell infiltration and promote tumor growth and metastasis [72,73,74]. In stark contrast, M1-like macrophages, human leukocyte antigen-DR^+^ (HLA-DR), retain antineoplastic activity through pro-inflammatory cytokine production, yet remain markedly underrepresented in established PDAC [72,73,75]. Rather than representing fixed cellular states, the M1/M2 balance reflects the dynamic cytokine and metabolic landscape of the TME, suggesting that repolarizing TAMs from an M2-like toward an M1-like phenotype could represent a rational therapeutic strategy to restore antitumoral immunity in PDAC. Importantly, semaphorins directly modulate TAM biology in PDAC, operating through multiple NRP and PLXN receptor axes to mainly reinforce an immunosuppressive myeloid landscape that supports tumor progression [76]. Class 3 semaphorins play a prominent role in shaping this compartment, particularly SEMA3D, which exemplifies direct modulation of TAM biology through a metabolic–immune axis downstream of PLXND1 signaling. Mechanistically, SEMA3D is produced by both tumor cells and infiltrating nerves and engages PLXND1 receptors on mutant-KRAS tumor cells to activate ADP-ribosylation factor 6 (ARF6), thereby elevating lactate production within PDAC cells. This tumor-derived lactate functions as a critical paracrine signal that reprograms infiltrating macrophages through G-protein-coupled receptor 132 (GPCR132) engagement, driving M2-like polarization and reinforcing the immunosuppressive myeloid landscape. Genetic ablation of SEMA3D in vivo prevented metastatic dissemination, prolonged survival, and substantially reduced M2 macrophage infiltration, underscoring how this class 3 semaphorin supports tumor progression through coordinated reprogramming of the TAM compartment [44]. Similarly, SEMA3C is highly expressed in KRAS^G12D^ PDAC cells and drives pro-tumoral macrophage polarization through regulation of autophagy and programmed death-ligand 1 (PD-L1) expression, correlating with M2 markers like CD163. SEMA3C inhibition reduces M2 polarization and tumor growth in xenograft models, identifying SEMA3C as a key regulator linking KRAS^G12D^ signaling to immunosuppressive TAM differentiation [76].

In basal-like PDAC, tumor-derived SEMA3A promotes macrophage recruitment and M2 polarization by involving NRP1 and PLXNA1. Colony-stimulating factor-1 receptor (CSF1R)-targeted macrophage depletion in SEMA3A^high^ tumors reverses M2 polarization, increases T-lymphocyte recruitment, and restores chemotherapy sensitivity, revealing NRP1-dependent SEMA3A-driven macrophage recruitment as a therapeutic vulnerability in basal-like PDAC [25]. Moreover, SEMA3A engages NRP1 to promote the sequestration of TAMs in hypoxic tumor regions through PLXNA1/A4, restricting macrophages to hypoxic niches [77]. Semaphorin-mediated TAM reprogramming extends beyond the primary tumor to the metastatic niche. In PDAC liver metastases, M2-polarized TAMs secrete SEMA5A, which interacts with PLXNB3 expressed on tumor cells, thereby modulating their metabolic state. Molecularly, SEMA5A-PLXNB3 signaling enhances aerobic glycolysis, driving the Warburg effect and promoting tumor cell proliferation and survival in the hepatic microenvironment. This interaction highlights the functional interplay between stromal immune cells and malignant cells in promoting liver metastatic outgrowth and suggests the SEMA5A-PLXNB3 pathway as a potential therapeutic vulnerability in unresectable PDAC with liver involvement [78]. The complexity of semaphorin–TAM interactions is further exemplified by SEMA4D, a pro-tumoral semaphorin implicated across multiple solid malignancies, including breast cancer [79], for which therapeutic targeting is being actively explored. In pancreatic cancer, specifically in the Rip1-Tag2 model of pancreatic neuroendocrine tumors, treatment with the anti-SEMA4D antibody (VX15/2503) reduced tumor growth and prolonged survival. However, SEMA4D blockade also promoted the accumulation of SEMA4D-positive macrophages at invasive tumor fronts, where these TAMs increased secretion of stromal cell-derived factor 1 (SDF1), thereby enhancing tumor cell invasion through C-X-C chemokine receptor type 4 (CXCR4) signaling [80]. These findings highlight potential limitations of SEMA4D blockade and suggest that PLXNB1, a major downstream receptor of SEMA4D, could represent an alternative therapeutic target in pancreatic cancer. Notably, PLXNB1 depletion promotes M1-like macrophage polarization in the immune microenvironment of triple-negative breast cancer [81], raising the possibility that PLXNB1 targeting might also influence TAM polarization in PDAC. Collectively, these findings position SEMAs and their cognate receptors as multilayered regulators of TAM recruitment, polarization, and function in PDAC, highlighting these pathways as potential immunotherapeutic targets.

While TAMs act as the principal architects of immunosuppression, CD8^+^ T cells are the effector arm most compromised by it. Their infiltration is sparse, and their function is eroded by exhaustion [61], which is particularly consequential given that a high CD8^+^ T cell density correlates with longer overall survival and disease-free survival [82]. Because checkpoint blockade relies on the presence of functional intratumoral T cells, this combined defect in infiltration and function explains why most PDAC cases remain refractory to these therapies [83,84]. Among the factors modulating these immune defects, axon guidance molecules, especially semaphorins and their receptors, are increasingly recognized as important regulators of the immune microenvironment [33,85].

Within this family, SEMA4D is a notable restrictor of immune infiltration in several solid tumor types, limiting the recruitment of tumor-reactive immune cells [86]. In PDAC, tumor-infiltrating lymphocytes are a major source of SEMA4D, which also promotes tumor cell invasiveness through the cancer epithelial PLXNB1 via RhoA/MAPK/Akt signaling, a pathway associated with lymph node metastasis and poor prognosis [46]. Consistent with this, in other tumor models, including Colon26 and ERBB2^+^ mammary carcinoma, antibody-mediated SEMA4D blockade enhanced monocyte and lymphocyte infiltration and augmented antitumor immunity, particularly when combined with immune checkpoint inhibition [86]. This rationale has led to the clinical evaluation of pepinemab, a humanized IgG4 anti-SEMA4D antibody, in PDAC in two trials in combination with immunotherapy: a phase 1b/2 trial (https://clinicaltrials.gov/study/NCT05102721) (accessed on 16 October 2026) testing it with the PD-L1 inhibitor avelumab as second-line therapy for metastatic pancreatic adenocarcinoma, and a phase 1 trial assessing its effects, alone or with immune checkpoint inhibitors, in resectable pancreatic cancer (https://clinicaltrials.gov/ct2/show/NCT03373188) (accessed on 16 October 2026) [87]. In contrast, recent multi-dataset analyses of PDAC patients have shown that SEMA4D expression is predominantly enriched in immune cells, with comparatively lower expression in malignant and stromal compartments. Interestingly, high immune cell-derived SEMA4D expression was associated with improved clinical outcomes, indicating a dichotomous role for SEMA4D in PDAC [88]. These observations highlight the context-dependent nature of SEMA4D biology and suggest that its immunomodulatory functions depend on the receptor-expressing cell populations it targets. Mechanistic studies are therefore needed to clarify these cell type-specific contributions, particularly with respect to immune checkpoint efficacy.

Alongside SEMA4D, another class 4 semaphorin, SEMA4B, has recently emerged as a critical regulator of PDAC immunosuppression. As already observed in lung adenocarcinoma [89], SEMA4B is significantly overexpressed in pancreatic cancer, where it enforces immune exclusion by selectively depleting multiple antitumor immune cell populations, including CD8^+^ T cells, while simultaneously enriching protumor cell populations, such as regulatory T cells. This immunomodulatory function, driven by promoter hypomethylation in early-stage disease, substantially correlates with poor clinical outcomes and identifies SEMA4B as a potential therapeutic target for reversing immune suppression in PDAC [90]. Collectively, semaphorins function as immunological gatekeepers in PDAC, regulating CD8^+^ T cell antitumor function. Targeting this semaphorin axis warrants investigation to enhance checkpoint inhibitor responsiveness and CD8^+^ T cell function.

Beyond TAMs and T cells, the role of semaphorin signaling in other immune populations of the tumor microenvironment, such as tumor-associated neutrophils [83,91], is being explored in other solid tumors. However, direct evidence in PDAC is currently lacking, and defining the role of semaphorin signaling in these underexplored immune populations may reveal new targets for therapeutic exploitation and expand the scope of semaphorin-directed immunomodulation.

### 3.3. Tumor Vasculature

A defining feature of PDAC is the complex remodeling of the vascular architecture within the desmoplastic microenvironment, characterized by aberrant angiogenesis coupled with vessel compression, poor perfusion and heterogeneous vascular distribution [92,93]. This vascular phenotype is also spatially heterogeneous, as human PDAC shows a hypovascular juxtatumoral stroma alongside a hypervascular adjacent normal rim, a pattern associated with poor patient prognosis [94]. Metabolic features add a further layer. Desmoplasia-mediated poor perfusion creates hypoxic regions in which HIF-1α upregulates glucose transporters and glycolytic enzymes, shifting cancer cells toward glycolysis [95]. This enhanced glycolysis sustains hypoxic cancer cells and fuels normoxic ones through shared metabolites, while also promoting immunosuppression and immunotherapy resistance [96]. Furthermore, elevated interstitial fluid pressure, together with a dense and fibrotic ECM, collapses intratumoral vessels, impairing perfusion and restricting the delivery and distribution of therapeutic agents, a phenomenon termed stromal resistance [97]. Human PDACs frequently exhibit increased expression of multiple pro-angiogenic factors and their cognate high-affinity receptors. Consistent with these findings, anti-angiogenic therapies have demonstrated significant antitumor activity in preclinical PDAC models. However, clinical trials evaluating anti-angiogenic strategies, predominantly through VEGF pathway blockade, have largely failed to demonstrate meaningful therapeutic benefit, highlighting the need for alternative therapeutic approaches that simultaneously target multiple regulators of tumor vasculature and its crosstalk with the TME [98,99]. Regarding this, SEMAs and PLXNs represent novel modulators of this aberrant vasculature and potential therapeutic targets. For instance, SEMA3A represents a critical regulator of pathological angiogenesis in pancreatic cancer. Systemic overexpression of SEMA3A in a mouse model of pancreatic neuroendocrine tumors (Rip1-Tag2) hampered tumor progression, reduced abnormal vasculature, and increased pericyte coverage by functioning as an endogenous inhibitor of angiogenesis [34]. Notably, SEMA3A, by inducing vessel normalization, inhibiting tumor hypoxia and a pro-invasive pathway in cancer cells, efficiently overcame the resistance induced by anti-angiogenic inhibitors in Rip1-Tag2 mice [35]. In addition, a rationally engineered NRP1-independent mutant of SEMA3A that selectively engages PLXNA4 provides a promising strategy for vascular normalization in PDAC. This mutant isoform of SEMA3A efficiently normalizes aberrant tumor vasculature, restores functional perfusion, inhibits hypoxia and suppresses both primary tumor growth and metastatic spread. Interestingly, vascular normalization achieved through mutant SEMA3A substantially enhances gemcitabine penetration and anticancer efficacy, providing a mechanistic basis for combining engineered semaphorin-based vascular normalization with conventional cytotoxic therapies to overcome PDAC treatment resistance [24]. In contrast to SEMA3A, semaphorin-5A (SEMA5A) operates with a pro-angiogenic role in PDAC, promoting pathological vascular remodeling through direct effects on endothelial cells. SEMA5A extracellular domain (SEMA5A-ECD), secreted by cancer cells, significantly enhances endothelial cell proliferation in vitro and substantially increases microvessel density within orthotopic PDAC tumors, driving enhanced angiogenesis despite paradoxically reducing primary tumor burden. These pro-angiogenic effects of secreted SEMA5A are mediated through upregulation of critical angiogenic molecules interleukin-8 (IL-8) and VEGF. Beyond its effects on endothelial cells, SEMA5A directly promotes a pro-invasive phenotype in PDAC cells by increasing ERK phosphorylation, thereby enhancing hepatic micrometastatic dissemination in vivo [100], or through M2 TAM-derived SEMA5A engaging PLXNB3 on PDAC cells to drive metastatic outgrowth via aerobic glycolysis [78]. This multifaceted pro-angiogenic and pro-metastatic role distinguishes SEMA5A from vessel-normalizing semaphorins, linking its increased signaling to the dysfunctional hypervascular phenotype and aggressive behavior of PDAC [47,100,101]. Another class-3 semaphorin that modulates PDAC vasculature is SEMA3E. While SEMA3E can exert anti-angiogenic effects through Plexin D1 in other cancer types [102], its overexpression in PDAC cells has instead been associated with increased CD31^+^ microvessel density and enhanced tumor growth, ultimately correlating with poorer patient survival [28]. This pro-angiogenic activity may, at least in part, reflect the ability of SEMA3E, particularly its 61 kDa cleaved form, to promote endothelial cell migration and growth through ERK1/2 activation, as shown in a breast cancer model of lung metastasis [103]. Overall, SEMA3E appears to promote tumor angiogenesis in PDAC, in contrast to the anti-angiogenic effects reported in other cancer types [104,105], although its precise role in this disease warrants more in-depth investigation. Finally, TAM-derived SEMA4D has also been implicated as a critical driver of angiogenesis in other types of cancer [106]. Importantly, in the Rip1-Tag2 model, anti-SEMA4D antibody treatment reduced tumor burden and extended survival without a classic antiangiogenic effect: vessel number, CD31^+^ area density, basement membrane deposition, and endothelial junction structure were unchanged. Instead, treatment normalized the tumor vasculature by altering pericyte coverage, with an increase in desmin^+^ and NG2^+^ pericytes, and this was accompanied by improved vascular integrity, as shown by reduced vascular leakage and a significant decrease in hemorrhagic tumors [80]. Recent studies have demonstrated that normalization of the tumor vasculature can enhance CD8^+^ tumor-infiltrating lymphocyte recruitment and function by restoring productive crosstalk between endothelial and immune cells, thereby improving responses to immunotherapy across multiple solid tumor types [107,108,109]. In this context, SEMAs-PLXNs signaling may represent an attractive therapeutic target in PDAC, as modulation of this pathway can promote the conversion of aberrant tumor vessels into a more stable and functional vasculature, potentially improving immune cell infiltration and activation, thereby enhancing the efficacy of immunotherapy.

### 3.4. Neural Remodeling

Beyond the desmoplastic stroma, the “cold” and immunosuppressive TME and the aberrant vasculature, PDAC is characterized by profound remodeling of its neural microenvironment, featuring aggressive perineural invasion (PNI), nerve hyperplasia and extensive tumor–nerve crosstalk that enhances neurotrophism and fuels tumor progression [110,111,112]. Despite their canonical roles in axon guidance, the contribution of SEMAs and PLXNs to tumor innervation remains poorly characterized [27]. Notably, SEMA3D and PLXND1 are emerging as critical orchestrators of pathological neural remodeling in PDAC by coordinating effects on tumor cells, nerves, and immune cells. Beyond its role within tumor cells, where SEMA3D engages PLXND1 to activate KRAS-dependent ARF6 signaling and drive glycolytic gene expression and lactate production via the Warburg effect [44], SEMA3D also acts in paracrine fashion on peripheral neurons. Specifically, SEMA3D secretion by pancreatic cancer cells activates PLXND1 on dorsal root ganglion neurons, establishing a paracrine signaling axis that enhances tumor cell migratory and invasive capacities [43,113]. This bidirectional tumor–nerve communication operates through reciprocal signaling: SEMA3D engagement of PLXND1 on neuronal cells promotes neurite outgrowth and neural hypertrophy within the TME while simultaneously amplifying tumor cell chemotaxis and invasion toward nerve fibers. Consistently, elevated expression of both SEMA3D and PLXND1 in human specimens, coupled with reduced spatial distance between tumor cells and nerves, strongly correlates with histologically confirmed PNI [27]. Furthermore, SEMA3D-driven metabolic reprogramming of PDAC cells through KRAS-dependent ARF6 signaling enhances lactate production, which engages GPCR132 on macrophages to promote protumor M2 polarization. Supporting this metabolic–immune signaling axis, multiplex immunohistochemistry revealed increased M2-polarized macrophages adjacent to SEMA3D-expressing nerves in human PDAC tissue [44,114]. Another semaphorin implicated in pancreatic neural remodeling is SEMA7A, which is produced by myofibroblasts to orchestrate neo-innervation within the pancreatic microenvironment. Mechanistically, TGF-β1-activated myofibroblasts upregulate and secrete SEMA7A, along with other neurotropic factors, in a transforming growth factor beta receptor 2-dependent manner, thereby promoting sympathetic nerve infiltration into inflamed and preneoplastic pancreatic tissue [59], where sympathetic signaling and associated adrenergic receptors contribute to PDAC initiation and progression [115]. Additionally, sympathetic signaling promotes CAF activation and SEMA3C expression [60], which enhances cancer stemness and correlates with reduced survival in PDAC patients [26]. Collectively, these findings reveal semaphorins and plexins as central architects of PDAC’s neural microenvironment, orchestrating tumor–nerve crosstalk across metabolism, immunity, and invasion, and positioning this axis as an emerging therapeutic opportunity.

## 4. The Broader Axon Guidance Network

The axon guidance landscape in PDAC extends beyond semaphorins and plexins, with additional molecular families activating distinct signaling pathways to promote cancer growth, metastatic dissemination and stromal remodeling. Netrin-1 (NTN1) exemplifies how these alternative guidance molecules reshape PDAC biology through coordinated signaling across the tumor epithelium, neural compartment, endothelium, and stromal microenvironment. NTN1 is upregulated in pancreatic epithelial cells by KRAS mutation and β-adrenergic signaling through MAPK activation, where it engages neogenin 1 (NEO1) on tumor cells to activate FAK and ERK pathways driving EMT, stemness, and invasion, while simultaneously engaging NEO1 on sympathetic neurons to promote axonal growth and branching [116]. NTN1 also confers apoptosis resistance and enhances the invasion of endothelial cells to support angiogenic remodeling [117]. However, NTN1 effects on the neural compartment are highly context- and receptor-dependent. During early pancreatic tumorigenesis, NTN1 is upregulated in metaplastic and pancreatic intraepithelial neoplasia (PanIN) lesions, promoting local sympathetic axon growth and branching through its neuronal receptor deleted in colorectal cancer (DCC). Rather than promoting tumor progression, this early neuroplastic response appears protective, as disruption of NTN1/DCC signaling accelerates PanIN formation and progression by increasing precancerous cell proliferation [118]. This stage-dependent role of NTN1 is further evident at metastatic sites, where PDAC-derived extracellular vesicles bearing NTN1 activate hepatic stellate cells and induce retinoic acid secretion, which in turn upregulates NTN1 in disseminated tumor cells through retinoic acid receptor/retinoid X receptor-E74-like factor 3 (RAR/RXR-ELF3) signaling, preparing the metastatic niche and promoting liver colonization [119]. The therapeutic potential of targeting NTN1 is further supported by recent clinical evidence, showing that NTN1 blockade combined with mFOLFIRINOX was well tolerated and associated with improved clinical outcomes in patients with locally advanced PDAC, with transcriptomic analyses revealing a reduction in EMT following treatment. Consistent with these findings, preclinical studies showed that NP137, an anti-NTN1 antibody, inhibited tumor cell migration in a NEO1-dependent manner, further supporting the role of NTN1-NEO1 signaling in tumor cell plasticity and dissemination [120]. Within the same family, Netrin-G1 (NetG1) marks a specialized CAF subset that reprograms glutamate/glutamine metabolism via protein kinase B/eukaryotic translation initiation factor 4E-binding protein 1, p38 mitogen-activated protein kinase/FOS-related antigen 1, and glutamine synthetase signaling while simultaneously suppressing NK cell activity; notably, NetG1 blockade reduces tumor burden in orthotopic models [121].

Beyond the netrin family, the SLIT-ROBO pathway constitutes another axon guidance system recurrently altered in PDAC. SLIT ligands and ROBO receptors harbor structural variations or mutations in approximately one-third of patients [122] and are frequently silenced by methylation [123], yet their functional role in pancreatic tumorigenesis remains poorly understood [124]. Emerging evidence, however, shows that epithelial ROBO2 acts as a pancreatic stroma suppressor by restraining tissue remodeling and tumor-promoting inflammation. Importantly, its loss triggers TGF- and Wnt signaling, which converts stromal cells into activated myofibroblasts, upregulates ROBO1, and accelerates collagen crosslinking. This loss also recruits CD3^+^ T cells and elevates immunosuppressive factors (e.g., IL-10), resulting in a protumor landscape where patients with low ROBO2 and high ROBO1 expression exhibit poorer survival outcomes [125]. Complementing these findings, SLIT2 also appears to function as a tumor-suppressive cue and is downregulated in pancreatic cancer cells [126]. Functionally, restoration of SLIT2 reinstates its repulsive guidance activity, limiting directed PDAC cell migration toward neural cells and along growing neurites, while also restricting endothelial cell migration. Consistently, restoring SLIT2 in vivo reduces tumor invasion, vascularization, and metastatic spread, whereas reduced SLIT2 expression is associated with greater lymph node involvement, supporting disruption of SLIT-ROBO signaling as a mechanism that facilitates PDAC dissemination [126]. Importantly, the effects of SLIT2 appear to depend critically on its cellular source. Within the stromal compartment, CAF-derived SLIT2 promotes the neural remodeling associated with PDAC progression by enhancing neurite outgrowth and stimulating Schwann cell migration and proliferation through modulation of N-cadherin/β-catenin signaling, thereby strengthening the stromal–neural connection [127]. Inhibition of SLIT2-ROBO signaling disrupts this interaction, highlighting a functional dichotomy whereby SLIT2 can either constrain tumor dissemination or promote a pro-neuroplastic microenvironment depending on its cellular and spatial context [124,127]. These findings demonstrate that axon guidance signaling in PDAC encompasses additional molecular families alongside semaphorins and plexins. Together, NTN1 and SLIT-ROBO signaling illustrate how other guidance systems act in a complementary and partially overlapping manner with the semaphorin–plexin axis, converging on the regulation of tumor progression, stromal remodeling, and immune exclusion through stage- and context-dependent mechanisms. As such, these pathways extend the framework established by SEMA/PLXN signaling and represent additional therapeutic nodes in pancreatic cancer biology.

## 5. Semaphorins and Plexins as Biomarkers and Targets for Preclinical Therapeutic Strategies in PDAC

### 5.1. Axon Guidance Molecules as Candidate Prognostic and Predictive Biomarkers in PDAC

The expression profiles of semaphorins and their plexin and neuropilin co-receptors have been investigated for their potential clinical value in the prognostic stratification of patients and in predicting biologically relevant phenomena such as PNI, immune infiltration, and immunotherapy responsiveness. An example is represented by a systematic multi-omics study, which integrated public TCGA and GEO cohorts to characterize the SEMA3 gene family in PDAC. Independent of conventional clinicopathological features, based on the stratification of SEMA3 expression, PDAC patients were clustered into two molecular subtypes with distinct survival outcomes and immune infiltration profiles. The nine-gene prognostic model was validated across an independent cohort, which confirmed its robustness and generalizability. The low-risk patients are characterized by a better immunotherapy response, with higher infiltration of CD8^+^ T cells, myeloid dendritic cells, and B cells, elevated immune checkpoint gene expression, and a higher tumor mutational burden. Conversely, the high-risk group showed a higher KRAS mutation frequency, associated with a worse prognosis. Furthermore, expression of the SEMA3 gene family revealed high heterogeneity across cell populations and tumor regions, as highlighted by the integration of single-cell RNA sequencing and spatial transcriptomics. Among all, SEMA3C showed the highest tumor specificity and the strongest association with poor prognosis, being mostly expressed in malignant epithelial cells and CAFs [128]. This heterogeneity indicates that bulk measurements tend to smooth out signals originating from different cellular compartments, thus limiting their interpretation at the tissue level. Also, these results show support for further validation before their potential implementation in clinical practice.

Similarly, annexin A2 (ANXA2) and SEMA3D have emerged as potential therapeutic targets and prognostic biomarkers of metastatic PDAC. In KPC (Kras^LSL-G12D/+^; Trp53^R172H/+^; Pdx1-Cre mouse models), ANXA2 promotes metastatic dissemination by inducing SEMA3D secretion, which activates signaling through PLXND1 expressed on tumor cells. Genetic depletion of either ANXA2 or SEMA3D reduces tumor cell invasiveness and metastatic spread. However, restoration of SEMA3D alone is insufficient to fully rescue the metastatic phenotype of ANXA2-deficient cells, suggesting that ANXA2 promotes metastasis through additional SEMA3D-independent mechanisms, highlighting how the redundancy of this network may weaken the predicted effect of the treatment. Consistent with these preclinical findings, high SEMA3D expression in primary PDAC is associated with more extensive metastatic disease and poorer survival compared with tumors exhibiting low SEMA3D expression [43]. The SEMA3D-PLXND1 signaling axis has also been implicated in perineural invasion, a hallmark of PDAC progression. Silencing either tumor-derived SEMA3D or PLXND1 reduces tumor innervation and metastatic dissemination in vivo whereas elevated SEMA3D/PLXND1 expression and increased proximity between tumor cells and nerves are associated with perineural invasion in human PDAC specimens [27]. Collectively, these findings identify the ANXA2-SEMA3D-PLXND1 signaling axis as an important regulator of PDAC dissemination and tumor–nerve interactions, with potential prognostic and therapeutic relevance.

Despite their potential clinical relevance, several methodological challenges remain, including the lack of standardized assays, predefined cutoff values, adjustment for tumor purity and clinical covariates, and independent validation in appropriate patient cohorts. Also, further evidence is needed to distinguish predictive from prognostic value and their associations with perineural invasion or immune infiltration.

### 5.2. Preclinical Therapeutic Strategies Targeting Semaphorins and Plexins Molecules in PDAC

Therapeutically, direct SEMA-PLXN-NRP signaling modulation through monoclonal antibodies represents the primary strategy currently in development. The monoclonal antibody N2E4 exemplifies this approach, specifically blocking NRP2-integrin β1 interaction via direct NRP2 engagement. By disrupting this interaction, N2E4 attenuates activation of the Focal Adhesion Kinase/Extracellular Signal-Regulated Kinase/Hypoxia-Inducible Factor-1α (FAK/ERK/HIF-1α) pathway and inhibits VEGF signal transduction. Consequently, it suppresses cancer cell proliferation, migration, invasion, and EMT, while reducing tumor growth and metastasis in vivo [129]. Because semaphorin signaling is governed by combinatorial and partially redundant ligand–receptor complexes, the effect of an in vivo intervention is often difficult to predict, and therapeutic efforts have therefore been directed at achieving receptor selectivity. A representative example involves engineered ligand variants, such as a particularly promising engineered SEMA3A mutant independent of NRP1 for its binding and signaling. This mutant SEMA3A binds its receptor PLXNA4 with greater affinity and biological activity than the wild-type protein, without triggering the NRP1-dependent signaling and its potentially associated adverse effects [77]. In syngeneic orthotopic PDAC mouse models, systemic administration of this mutant isoform led to tumor vasculature normalization, tumor growth and metastasis inhibition, and enhanced chemotherapy delivery and efficacy [24]. Another approach implicates an uncleavable variant of SEMA3E (Uncl-SEMA3E), which binds PLXND1 without inducing PLXND1-ERBB2 interaction and the related pro-metastatic signaling. Uncl-SEMA3E blocks tumor cell metastatic ability by competing with endogenous p61-SEMA3E, as well as acting as an anti-angiogenic factor through PLXND1 signaling. In the Rip1-Tag2 pancreatic neuroendocrine tumor model, local administration of Uncl-SEMA3E markedly reduced tumor burden and vascular area/coverage, increasing endothelial apoptosis and hypoxia, with an anti-angiogenic effect [130] (Table 1). It is noteworthy to highlight that to date, these interventions have been assessed in preclinical models, and their translational potential has not yet been established in clinical settings. Moreover, most of the evidence reviewed here derives from genetic approaches, for which a corresponding selective pharmacological tool is not always available.

## 6. Therapeutic Clinical Trials Targeting Semaphorins and Plexins in PDAC

Given the therapeutic evidence outlined above, semaphorins and their receptors are now the subject of promising clinical trials in PDAC [131]. In this regard, an ongoing Phase 1 clinical trial (https://clinicaltrials.gov/ct2/show/NCT03373188) (accessed on 16 October 2026) is evaluating pepinemab, a monoclonal antibody targeting SEMA4D, which has demonstrated clinical efficacy in other solid malignancies [87,132], including pancreatic neuroendocrine tumors [80]. The study examines the safety, tolerability, pharmacokinetics, pharmacodynamics, and antitumor efficacy of pepinemab administered in combination with ipilimumab or nivolumab in patients with advanced solid tumors, including stage I-III PDAC. The overarching objective of this trial is to enhance immunotherapy responsiveness and clinical efficacy in PDAC patients after 5FU or gemcitabine-based first-line treatment by leveraging the immunomodulatory properties of SEMA4D blockade to augment checkpoint inhibitor activity within the tumor microenvironment. In addition, a separate Phase1b/2 clinical trial aims to assess the safety, tolerability and efficacy of pepinemab in combination with avelumab in metastatic PDAC patients, after first-line chemotherapy (https://clinicaltrials.gov/study/NCT05102721) (accessed on 16 October 2026). Another strategy is provided by a non-randomized Phase 1 study, which tested the cyclic peptide CEND-1, binding NRP1 and αV integrins expressed on the cell surface, aiming to enhance the penetration of anticancer drugs into neoplastic pancreatic tissue. In this trial, CEND-1 was combined with standard treatment with nab-paclitaxel and gemcitabine. Even in this case, its safety, tolerability and biological efficacy were evaluated (https://clinicaltrials.gov/ct2/show/NCT03517176) (accessed on 16 October 2026).

These ongoing trials (Table 2) underscore the therapeutic versatility of semaphorin pathway targeting in PDAC, encompassing both immunomodulation and enhanced drug delivery, and position this axis as a rational and promising avenue for clinical advancement. However, the therapeutic rationale remains primarily combination-specific rather than indicative of broad monotherapy efficacy, including the enhancement of chemotherapy delivery to tumor tissue and the modulation of the TME to increase responsiveness to immunotherapy.

## 7. Conclusions and Future Perspectives

A deeper understanding of the SEMA/PLXN signaling network in PDAC is limited by various aspects. Firstly, their functional redundancy and ligand–receptor plasticity represent a major obstacle to single-target therapies [133]. Also, semaphorins and plexins exert either tumor promoter- or suppressor-like effects depending on the cancer type, subtype, and microenvironmental context [134]. Future work will require the integration of multiple levels of analysis to overcome current limitations. In this respect, artificial intelligence and multi-omics approaches are increasingly being employed in oncology to identify resistant subclones, define the spatial immune architectures, and investigate potential biomarkers and therapeutic targets in tumors [135,136]. Applied to the SEMA/PLXN axis, such strategies could facilitate the selection of therapies targeting multiple nodes of the axon guidance network.

Recent semaphorin-focused studies combining bulk, single-cell and spatial transcriptomics in PDAC [128] have provided important insights into the SEMA/PLXN signaling components across the different compartments of the tumor microenvironment and their spatial organization, while highlighting their potential as biomarkers for patient stratification and therapeutic response. For instance, this integrated approach provides compelling evidence further confirming the critical role of SEMA3C in PDAC progression and immune microenvironment modulation [76].

For future applications, ligand–receptor inference tools such as CellChat or CellPhoneDB [137,138] could be applied to PDAC single-cell and spatial data to quantify which SEMA/PLXN interactions dominate between tumor cells and the cells populating the TME. Also, spatial transcriptomics could be used to determine whether SEMA/PLXN-high niches co-localize with specific regions in PDAC tissues. In addition, machine learning models that predict treatment response from single-cell transcriptomic profiles, such as Beyondcell [139], can prioritize therapeutic options for individual tumors or cell subpopulations. Although most of these tools were developed and benchmarked in other tumor types, they could be adapted to PDAC to test whether tumor subpopulations with high SEMA/PLXN activity display distinct predicted drug sensitivities.

Overall, computational and multi-omics approaches are powerful tools for identifying candidate biomarkers and therapeutic targets within the SEMA/PLXN network, but their predictions must be validated in experimental models. As discussed in the previous sections, inhibitors and modulators of this signaling axis are already available or under investigation, and a more comprehensive analysis of the crosstalk between SEMA/PLXN and other axon guidance cues could reveal further strategies. Challenges remain, including the context-dependent roles of individual semaphorins and the limited availability of selective inhibitors. For instance, SEMA4D, as discussed in this review, illustrates the first challenge, as it promotes PDAC invasiveness and restricts antitumor immune infiltration [46,86], yet its high expression in immune cells correlates with improved patient outcomes [88], suggesting that its effects depend on the receptor-expressing cell type it targets.

However, these obstacles are not insurmountable, and agents such as the anti-SEMA4D antibody pepinemab are already in clinical development. Other axon guidance cues, including NTN1 [120] and SLIT–ROBO [124] signaling, as well as EPH/Ephrin [140], also contribute to PDAC microenvironmental dysregulation and tumor progression. Thus, therapeutic strategies targeting SEMA-PLXN pathways, either alone or in combination with other axon guidance signaling networks, may provide novel opportunities to disrupt tumor-promoting crosstalk and improve therapeutic responses in PDAC. Taken together, a systematic and context-aware targeting of the SEMA/PLXN axis, guided by multi-omics discovery with experimental validation and combination strategies, may uncover new vulnerabilities and enable more durable, rational therapies in PDAC.

## Figures and Tables

**Table 1:** Therapeutic strategies targeting Semaphorins and Plexins signaling pathways in pancreatic tumors.

Therapeutic Strategy	Targeted Axis	Mechanism	Effects	Experimental Model
Monoclonal antibody anti-NRP2 (N2E4)	NRP2-integrin β1	It blocks the interaction NRP2-integrin β1, reducing the FAK/ERK/HIF-1α pathway and inhibiting VEGF signal transduction	Suppression of proliferation, migration, invasion, and EMT; reduction in tumoral growth and metastasis	PDAC cell lines; pancreatic cancer xenografts
Engineered SEMA3A mutant NRP1-independent	SEMA3A-PLXNA4	It binds PLXNA4 with high affinity and inhibits Rap-1 pathway in endothelial cells	Normalization of the tumor vasculature; inhibition of tumor growth and metastasis; improvement of chemotherapy delivery and efficacy	Syngeneic orthotopic PDAC mouse models
Uncleavable SEMA3E variant	SEMA3E-PLXND1	It binds PLXND1 without inducing the PLXND1-ERBB2 interaction	Suppression of metastasis; reduction in tumor burden, vascular area/coverage; increase in endothelial apoptosis and hypoxia	Rip1-Tag2 pancreatic neuroendocrine tumor model

**Table 2:** Therapeutic clinical trials targeting Semaphorins and Plexins in PDAC.

ClinicalTrials.Gov ID	Drug	Target	Phase and Population	Enrolled Patients (*n*)
NCT03373188	Pepinemab ± ipilimumab or nivolumab	SEMA4D	Phase 1 clinical study in PDAC and colorectal cancer patients	10
NCT05102721	Pepinemab in combination with avelumab	SEMA4D	Phase 1b/2 clinical study in metastatic PDAC patients, after 5FU or gemcitabine based first line treatment	48 estimated
NCT03517176	Cyclic peptide (CEND-1) in combination with nabpaclitaxel and gemcitabine	NRP1-αV integrins	Phase 1 clinical study in metastatic PDAC patients, in combination with standard treatment undergoing combination therapy with nab-paclitaxel and gemcitabine	30

## Data Availability

No new data were created or analyzed in this study. Data sharing is not applicable to this article.

## References

[1] Park W., Chawla A., O’Reilly E.M. (2021). Pancreatic Cancer: A Review. JAMA.

[2] Stoop T.F., Javed A.A., Oba A., Koerkamp B.G., Seufferlein T., Wilmink J.W., Besselink M.G. (2025). Pancreatic Cancer. Lancet.

[3] Yu J., Cao Y., Xia F., Lan Q., Zhou J., Wang Z., Ren S. (2026). Diagnostic Performance of Multislice Spiral Computed Tomography in Differentiating Pancreatic Acinar Cell Carcinoma from Pancreatic Ductal Adenocarcinoma. Abdom. Radiol..

[4] Costamagna A., Ghiglione N., Martini M. (2025). The Molecular Logic of Early Metastasis in Pancreatic Cancer: Crosstalk between Tumor and Microenvironment. Front. Cell Dev. Biol..

[5] Shah A., Ganguly K., Rauth S., Sheree S.S., Khan I., Ganti A.K., Ponnusamy M.P., Kumar S., Jain M., Batra S.K. (2024). Unveiling the Resistance to Therapies in Pancreatic Ductal Adenocarcinoma. Drug Resist. Updat..

[6] Malla M., Sakr Y., Gupta G., Akce M., El-Rayes B. (2025). Emerging Therapeutic Advancements in Pancreatic Cancer: A Contemporary Review. Discov. Oncol..

[7] Mannucci A., Goel A. (2026). Advances in Pancreatic Cancer Early Diagnosis, Prevention, and Treatment: The Past, the Present, and the Future. CA Cancer J. Clin..

[8] Moss R.A., Lee C. (2010). Current and Emerging Therapies for the Treatment of Pancreatic Cancer. OncoTargets Ther..

[9] Rozengurt E., Eibl G. (2026). Pancreatic Cancer: Molecular Pathogenesis and Emerging Therapeutic Strategies. Signal. Transduct. Target Ther..

[10] Wolpin B.M., Park W., Garrido-Laguna I., Spira A., Starodub A., Sommerhalder D., Punekar S.R., Barve M., Pelster M., Herzberg B. (2026). Daraxonrasib in Previously Treated Advanced RAS-Mutated Pancreatic Cancer. N. Engl. J. Med..

[11] O’Reilly E.M., Wainberg Z.A., Hendifar A.E., Borad M.J., Pietrantonio F., Pant S., Hammel P., Cremolini C., Manji G.A., Oberstein P.E. (2026). Daraxonrasib or Chemotherapy in Previously Treated Metastatic Pancreatic Cancer. N. Engl. J. Med..

[12] Aronchik I., Kar S., Zhuang Y., Ahler E., Lai L.P., Seshadri V., Yang Y.C., Bulle A., Menard M., Nayak B. (2026). Acquired Resistance to the RAS(ON) Multi-Selective Inhibitor Daraxonrasib Guides Rational Combination Therapy Strategies in Pancreatic Cancer. Nat. Med..

[13] Dilly J., Hoffman M.T., Abbassi L., Li Z., Paradiso F., Parent B.D., Hennessey C.J., Jordan A.C., Morgado M., Dasgupta S. (2024). Mechanisms of Resistance to Oncogenic KRAS Inhibition in Pancreatic Cancer. Cancer Discov..

[14] Ciulla C., Luchini C. (2024). Genomic Determinants of Biological Aggressiveness and Poor Prognosis of Pancreatic Cancers: *KRAS* and Beyond. Expert Rev. Mol. Diagn..

[15] Kung H.-C., Zheng K.W., Zimmerman J.W., Zheng L. (2025). The Tumour Microenvironment in Pancreatic Cancer—New Clinical Challenges, but More Opportunities. Nat. Rev. Clin. Oncol..

[16] Gomes B.M., Xavier P.L.P. (2026). The Tumor Microenvironment in Pancreatic Cancer: From Composition to Therapeutic Targeting. Biochem. Soc. Trans..

[17] Chen M.-M., Gao Q., Ning H., Chen K., Gao Y., Yu M., Liu C.-Q., Zhou W., Pan J., Wei L. (2025). Integrated Single-Cell and Spatial Transcriptomics Uncover Distinct Cellular Subtypes Involved in Neural Invasion in Pancreatic Cancer. Cancer Cell.

[18] Li Y., Ding Z., Cheng T., Hu Y., Zhong F., Ren S., Wang S. (2025). Single-Cell Transcriptomics Analysis Reveals Dynamic Changes and Prognostic Signature in Tumor Microenvironment of PDAC. Sci. Rep..

[19] Fernández-Nogueira P., Linzoain-Agos P., Cueto-Remacha M., De la Guia-Lopez I., Recalde-Percaz L., Parcerisas A., Gascon P., Carbó N., Gutierrez-Uzquiza A., Fuster G. (2024). Role of Semaphorins, Neuropilins and Plexins in Cancer Progression. Cancer Lett..

[20] Toledano S., Neufeld G. (2023). Plexins as Regulators of Cancer Cell Proliferation, Migration, and Invasivity. Cancers.

[21] Chen S., Chen B., Li G., Liu H., Chen Q., Chen J., Xu Y., Aipire A., Li J. (2026). Slc1a2-Mediated Glutamate Transport Promotes Dendritic Cell Immunity through Sema3A-Regulated Cytoskeletal Remodeling and Migration. Int. J. Biol. Sci..

[22] Chang Y., Li L., Zhang L., Guo X., Feng Z., Zhou J., Zhou S., Feng G., Han F., Huang W. (2016). Plexin-B1 Indirectly Affects Glioma Invasiveness and Angiogenesis by Regulating the RhoA/Avβ3 Signaling Pathway and SRPK1. Tumour. Biol..

[23] Dhall A., Taniyama D., Elloumi F., Luna A., Varma S., Kumar S., Escobedo L., Sun Y., Aladjem M.I., Redon C.E. (2025). Bimodal Genomic Approach Predicting Semaphorin 7A (SEMA7A) as Prognostic Biomarker in Adrenocortical Carcinoma. Cancers.

[24] Gioelli N., Maione F., Camillo C., Ghitti M., Valdembri D., Morello N., Darche M., Zentilin L., Cagnoni G., Qiu Y. (2018). A Rationally Designed NRP1-Independent Superagonist SEMA3A Mutant Is an Effective Anticancer Agent. Sci. Transl. Med..

[25] Lupo F., Pezzini F., Pasini D., Fiorini E., Adamo A., Veghini L., Bevere M., Frusteri C., Delfino P., D’agosto S. (2024). Axon Guidance Cue SEMA3A Promotes the Aggressive Phenotype of Basal-like PDAC. Gut.

[26] Tomizawa S., Takano S., Eto R., Takayashiki T., Kuboki S., Ohtsuka M. (2023). Semaphorin 3 C Enhances Putative Cancer Stemness and Accelerates Peritoneal Dissemination in Pancreatic Cancer. Cancer Cell Int..

[27] Jurcak N.R., Rucki A.A., Muth S., Thompson E., Sharma R., Ding D., Zhu Q., Eshleman J.R., Anders R.A., Jaffee E.M. (2019). Axon Guidance Molecules Promote Perineural Invasion and Metastasis of Orthotopic Pancreatic Tumors in Mice. Gastroenterology.

[28] Yong L.-K., Lai S., Liang Z., Poteet E., Chen F., van Buren G., Fisher W., Mo Q., Chen C., Yao Q. (2016). Overexpression of Semaphorin-3E Enhances Pancreatic Cancer Cell Growth and Associates with Poor Patient Survival. Oncotarget.

[29] Shen S., Qian X., Shi Z., Wu Y., Zhou Z., Nie S., Yu Y., Zhou S., Liu K., Zhou Q. (2026). ALDH1A3 Promotes the Lung Metastatic Organotropism of Pancreatic Ductal Adenocarcinoma through Semaphorin Signaling. iScience.

[30] Tam K.J., Hui D.H.F., Lee W.W., Dong M., Tombe T., Jiao I.Z.F., Khosravi S., Takeuchi A., Peacock J.W., Ivanova L. (2017). Semaphorin 3 C Drives Epithelial-to-Mesenchymal Transition, Invasiveness, and Stem-like Characteristics in Prostate Cells. Sci. Rep..

[31] Wang J.-J., Xie L., Wang X., Chen X.-Y., Xu X., Zhai P.-P., Xu Z.-Z., Chen K. (2026). SEMA4F Regulates the Malignant Phenotype of Hepatocellular Carcinoma Cells Through the PI3K-Akt Pathway. J. Hepatocell. Carcinoma.

[32] Zhang Y., Zuo A., Ba Y., Liu S., Chen J., Yang S., Weng S., Chen Y., Xu H., Luo P. (2025). Cancer-Associated Fibroblast-Derived SEMA3C Facilitates Colorectal Cancer Liver Metastasis via NRP2-Mediated MAPK Activation. Proc. Natl. Acad. Sci. USA.

[33] Jiang J., Zhang F., Wan Y., Fang K., Yan Z.-D., Ren X.-L., Zhang R. (2022). Semaphorins as Potential Immune Therapeutic Targets for Cancer. Front. Oncol..

[34] Maione F., Molla F., Meda C., Latini R., Zentilin L., Giacca M., Seano G., Serini G., Bussolino F., Giraudo E. (2009). Semaphorin 3A Is an Endogenous Angiogenesis Inhibitor That Blocks Tumor Growth and Normalizes Tumor Vasculature in Transgenic Mouse Models. J. Clin. Investig..

[35] Maione F., Capano S., Regano D., Zentilin L., Giacca M., Casanovas O., Bussolino F., Serini G., Giraudo E. (2012). Semaphorin 3A Overcomes Cancer Hypoxia and Metastatic Dissemination Induced by Antiangiogenic Treatment in Mice. J. Clin. Investig..

[36] Gu C., Limberg B.J., Whitaker G.B., Perman B., Leahy D.J., Rosenbaum J.S., Ginty D.D., Kolodkin A.L. (2002). Characterization of Neuropilin-1 Structural Features That Confer Binding to Semaphorin 3A and Vascular Endothelial Growth Factor 165. J. Biol. Chem..

[37] Miao H.Q., Soker S., Feiner L., Alonso J.L., Raper J.A., Klagsbrun M. (1999). Neuropilin-1 Mediates Collapsin-1/Semaphorin III Inhibition of Endothelial Cell Motility: Functional Competition of Collapsin-1 and Vascular Endothelial Growth Factor-165. J. Cell Biol..

[38] Serini G., Bussolino F., Maione F., Giraudo E. (2013). Class 3 Semaphorins: Physiological Vascular Normalizing Agents for Anti-Cancer Therapy. J. Intern. Med..

[39] Wang Y., He H., Srivastava N., Vikarunnessa S., Chen Y., Jiang J., Cowan C.W., Zhang X. (2012). Plexins Are GTPase-Activating Proteins for Rap and Are Activated by Induced Dimerization. Sci. Signal..

[40] Hao J., Yu J.S. (2018). Semaphorin 3C and Its Receptors in Cancer and Cancer Stem-Like Cells. Biomedicines.

[41] Xu X., Zhao Z., Guo S., Li J., Liu S., You Y., Ni B., Wang H., Bie P. (2017). Increased Semaphorin 3c Expression Promotes Tumor Growth and Metastasis in Pancreatic Ductal Adenocarcinoma by Activating the ERK1/2 Signaling Pathway. Cancer Lett..

[42] Liu D., Li J., Qi F., You H. (2022). Semaphorins and Their Receptors in Pancreatic Cancer: Mechanisms and Therapeutic Opportunities. Front. Oncol..

[43] Foley K., Rucki A.A., Xiao Q., Zhou D., Leubner A., Mo G., Kleponis J., Wu A.A., Sharma R., Jiang Q. (2015). Semaphorin 3D Autocrine Signaling Mediates the Metastatic Role of Annexin A2 in Pancreatic Cancer. Sci. Signal..

[44] Thielman N.R.J., Funes V., Davuluri S., Ibanez H.E., Sun W.-C., Fu J., Li K., Muth S., Pan X., Fujiwara K. (2024). Semaphorin 3D Promotes Pancreatic Ductal Adenocarcinoma Progression and Metastasis through Macrophage Reprogramming. Sci. Adv..

[45] Gu C., Yoshida Y., Livet J., Reimert D.V., Mann F., Merte J., Henderson C.E., Jessell T.M., Kolodkin A.L., Ginty D.D. (2005). Semaphorin 3E and Plexin-D1 Control Vascular Pattern Independently of Neuropilins. Science.

[46] Kato S., Kubota K., Shimamura T., Shinohara Y., Kobayashi N., Watanabe S., Yoneda M., Inamori M., Nakamura F., Ishiguro H. (2011). Semaphorin 4D, a Lymphocyte Semaphorin, Enhances Tumor Cell Motility through Binding Its Receptor, plexinB1, in Pancreatic Cancer. Cancer Sci..

[47] Saxena S., Hayashi Y., Wu L., Awaji M., Atri P., Varney M.L., Purohit A., Rachagani S., Batra S.K., Singh R.K. (2018). Pathological and Functional Significance of Semaphorin-5A in Pancreatic Cancer Progression and Metastasis. Oncotarget.

[48] Saxena S., Prajapati D.R., Goel P., Tomar B., Hayashi Y., Atri P., Rachagani S., Grandgenett P.M., Hollingsworth M.A., Batra S.K. (2021). Plexin-B3 Regulates Cellular Motility, Invasiveness, and Metastasis in Pancreatic Cancer. Cancers.

[49] Hung Y.-H., Hsu S.-H., Hou Y.-C., Chu P.-Y., Su Y.-Y., Shan Y.-S., Hung W.-C., Chen L.-T. (2022). Semaphorin 6C Suppresses Proliferation of Pancreatic Cancer Cells via Inhibition of the AKT/GSK3/β-Catenin/Cyclin D1 Pathway. Int. J. Mol. Sci..

[50] Jin G., Hong W., Guo Y., Bai Y., Chen B. (2020). Molecular Mechanism of Pancreatic Stellate Cells Activation in Chronic Pancreatitis and Pancreatic Cancer. J. Cancer.

[51] Schnittert J., Bansal R., Prakash J. (2019). Targeting Pancreatic Stellate Cells in Cancer. Trends Cancer.

[52] Miyabayashi K., Ijichi H., Fujishiro M. (2022). The Role of the Microbiome in Pancreatic Cancer. Cancers.

[53] Erez N., Truitt M., Olson P., Hanahan D. (2010). Cancer-Associated Fibroblasts Are Activated in Incipient Neoplasia to Orchestrate Tumor-Promoting Inflammation in an NF-κB-Dependent Manner. Cancer Cell.

[54] Biffi G., Oni T.E., Spielman B., Hao Y., Elyada E., Park Y., Preall J., Tuveson D.A. (2019). IL1-Induced JAK/STAT Signaling Is Antagonized by TGFβ to Shape CAF Heterogeneity in Pancreatic Ductal Adenocarcinoma. Cancer Discov..

[55] Öhlund D., Handly-Santana A., Biffi G., Elyada E., Almeida A.S., Ponz-Sarvise M., Corbo V., Oni T.E., Hearn S.A., Lee E.J. (2017). Distinct Populations of Inflammatory Fibroblasts and Myofibroblasts in Pancreatic Cancer. J. Exp. Med..

[56] Elyada E., Bolisetty M., Laise P., Flynn W.F., Courtois E.T., Burkhart R.A., Teinor J.A., Belleau P., Biffi G., Lucito M.S. (2019). Cross-Species Single-Cell Analysis of Pancreatic Ductal Adenocarcinoma Reveals Antigen-Presenting Cancer-Associated Fibroblasts. Cancer Discov..

[57] Caligiuri G., Tuveson D.A. (2023). Activated Fibroblasts in Cancer: Perspectives and Challenges. Cancer Cell.

[58] Chen Y.-I., Tien S.-C., Ko Y.-L., Chang C.-C., Hsu M.-F., Chien H.J., Peng H.-Y., Jeng Y.-M., Tien Y.-W., Chang Y.-T. (2024). SEMA7A-Mediated Juxtacrine Stimulation of IGFBP-3 Upregulates IL-17RB at Pancreatic Cancer Invasive Front. Cancer Gene Ther..

[59] Nigri J., Lan W., Fung M.L., Kayser C., Deschênes A., Hinds J., Kaushalya S., Pawlak S.A., Thalappillil J.S., Nadella S. (2026). Myofibroblasts Induce Neuroplasticity to Promote Pancreatic Inflammation and Cancer Progression. Cancer Discov..

[60] Sattler A.L., Diba P., Hawthorne K., Pelz C., Grieco J., Korzun T., Chong B., Kuykendall M.J., Sears R.C., Marks D.L. (2026). Sympathetic Nerve–Fibroblast Crosstalk Drives Nerve Injury, Fibroblast Activation, and Matrix Remodeling in Pancreatic Cancer. JCI Insight.

[61] Bräutigam K., Skok K., Szymonski K., Rift C.V., Karamitopoulou E. (2025). Tumor Immune Microenvironment in Pancreatic Ductal Adenocarcinoma Revisited-Exploring the “Space”. Cancer Lett..

[62] Principe D.R., Korc M., Kamath S.D., Munshi H.G., Rana A. (2021). Trials and Tribulations of Pancreatic Cancer Immunotherapy. Cancer Lett..

[63] Falcomatà C., Bärthel S., Schneider G., Rad R., Schmidt-Supprian M., Saur D. (2023). Context-Specific Determinants of the Immunosuppressive Tumor Microenvironment in Pancreatic Cancer. Cancer Discov..

[64] Yang J., Zhang Q., Wang J., Lou Y., Hong Z., Wei S., Sun K., Wang J., Chen Y., Sheng J. (2022). Dynamic Profiling of Immune Microenvironment during Pancreatic Cancer Development Suggests Early Intervention and Combination Strategy of Immunotherapy. EBioMedicine.

[65] Pan D., Li X., Qiao X., Wang Q. (2025). Immunosuppressive Tumor Microenvironment in Pancreatic Cancer: Mechanisms and Therapeutic Targets. Front. Immunol..

[66] Ju Y., Xu D., Liao M.-M., Sun Y., Bao W.-D., Yao F., Ma L. (2024). Barriers and Opportunities in Pancreatic Cancer Immunotherapy. npj Precis. Oncol..

[67] Li D., Liu Y., Lan R., Pillarisetty V.G., Zhang X., Liu Y.-Z. (2026). Neoadjuvant Therapy-Induced Remodeling of Tumor Immune Microenvironment in Pancreatic Ductal Adenocarcinoma: A Spatial and Digital Pathology Analysis. Virchows Arch..

[68] Zwart E.S., van Ee T., Doppenberg D., Farina A., Wilmink J.W., Versteijne E., Busch O.R., Besselink M.G., Meijer L.L., van Kooyk Y. (2023). The Immune Microenvironment after Neoadjuvant Therapy Compared to Upfront Surgery in Patients with Pancreatic Cancer. J. Cancer Res. Clin. Oncol..

[69] Yoshikawa K., Mitsunaga S., Kinoshita T., Konishi M., Takahashi S., Gotohda N., Kato Y., Aizawa M., Ochiai A. (2012). Impact of Tumor-Associated Macrophages on Invasive Ductal Carcinoma of the Pancreas Head. Cancer Sci..

[70] Santoni M., Bracarda S., Nabissi M., Massari F., Conti A., Bria E., Tortora G., Santoni G., Cascinu S. (2014). CXC and CC Chemokines as Angiogenic Modulators in Nonhaematological Tumors. Biomed. Res. Int..

[71] Pratt H.G., Steinberger K.J., Mihalik N.E., Ott S., Whalley T., Szomolay B., Boone B.A., Eubank T.D. (2021). Macrophage and Neutrophil Interactions in the Pancreatic Tumor Microenvironment Drive the Pathogenesis of Pancreatic Cancer. Cancers.

[72] Murray P.J. (2017). Macrophage Polarization. Annu. Rev. Physiol..

[73] Väyrynen S.A., Zhang J., Yuan C., Väyrynen J.P., Dias Costa A., Williams H., Morales-Oyarvide V., Lau M.C., Rubinson D.A., Dunne R.F. (2021). Composition, Spatial Characteristics, and Prognostic Significance of Myeloid Cell Infiltration in Pancreatic Cancer. Clin. Cancer Res..

[74] Tabe S., Takeuchi K., Aoshima K., Okumura A., Yamamoto Y., Yanagisawa K., Eto R., Matsuo M., Ueno Y., Konishi T. (2025). A Pancreatic Cancer Organoid Incorporating Macrophages Reveals the Correlation between the Diversity of Tumor-Associated Macrophages and Cancer Cell Survival. Biomaterials.

[75] Mercanti L., Sindaco M., Mazzone M., Di Marcantonio M.C., Piscione M., Muraro R., Mincione G. (2023). PDAC, the Influencer Cancer: Cross-Talk with Tumor Microenvironment and Connected Potential Therapy Strategies. Cancers.

[76] Zhang D., Lindstrom A., Kim E.J., Hwang C.-I., Hall M.L., Lin T.-Y., Li Y. (2022). SEMA3C Supports Pancreatic Cancer Progression by Regulating the Autophagy Process and Tumor Immune Microenvironment. Front. Oncol..

[77] Casazza A., Laoui D., Wenes M., Rizzolio S., Bassani N., Mambretti M., Deschoemaeker S., Van Ginderachter J.A., Tamagnone L., Mazzone M. (2013). Impeding Macrophage Entry into Hypoxic Tumor Areas by Sema3A/Nrp1 Signaling Blockade Inhibits Angiogenesis and Restores Antitumor Immunity. Cancer Cell.

[78] Wang K., He M., Fan X., Zhou J., Yang J., Wang L., Zhao Z., Dai C., Zhang Z. (2023). SEMA5A-PLXNB3 Axis Promotes PDAC Liver Metastasis Outgrowth through Enhancing the Warburg Effect. J. Immunol. Res..

[79] Jiang H., Chen C., Sun Q., Wu J., Qiu L., Gao C., Liu W., Yang J., Jun N., Dong J. (2016). The Role of Semaphorin 4D in Tumor Development and Angiogenesis in Human Breast Cancer. Onco Targets Ther..

[80] Zuazo-Gaztelu I., Pàez-Ribes M., Carrasco P., Martín L., Soler A., Martínez-Lozano M., Pons R., Llena J., Palomero L., Graupera M. (2019). Antitumor Effects of Anti-Semaphorin 4D Antibody Unravel a Novel Proinvasive Mechanism of Vascular-Targeting Agents. Cancer Res..

[81] Franzolin G., Brundu S., Cojocaru C.F., Curatolo A., Ponzo M., Mastrantonio R., Mihara E., Kumanogoh A., Suga H., Takagi J. (2024). PlexinB1 Inactivation Reprograms Immune Cells in the Tumor Microenvironment, Inhibiting Breast Cancer Growth and Metastatic Dissemination. Cancer Immunol. Res..

[82] Orhan A., Vogelsang R.P., Andersen M.B., Madsen M.T., Hölmich E.R., Raskov H., Gögenur I. (2020). The Prognostic Value of Tumour-Infiltrating Lymphocytes in Pancreatic Cancer: A Systematic Review and Meta-Analysis. Eur. J. Cancer.

[83] Li H.-B., Yang Z.-H., Guo Q.-Q. (2021). Immune Checkpoint Inhibition for Pancreatic Ductal Adenocarcinoma: Limitations and Prospects: A Systematic Review. Cell Commun. Signal..

[84] Giurini E.F., Ralph O., Pappas S.G., Gupta K.H. (2025). Looking Beyond Checkpoint Inhibitor Monotherapy: Uncovering New Frontiers for Pancreatic Cancer Immunotherapy. Mol. Cancer Ther..

[85] Liu Z., Wu Y., Xu P., Xu H., Yin K., Wang S. (2026). Semaphorins: From CD8^+^ T Cells to Cancer Immunotherapy. Biomark. Res..

[86] Evans E.E., Jonason A.S., Bussler H., Torno S., Veeraraghavan J., Reilly C., Doherty M.A., Seils J., Winter L.A., Mallow C. (2015). Antibody Blockade of Semaphorin 4D Promotes Immune Infiltration into Tumor and Enhances Response to Other Immunomodulatory Therapies. Cancer Immunol. Res..

[87] Rossi A.J., Khan T.M., Hong H., Lesinski G.B., Wu C., Hernandez J.M. (2021). Pepinemab (Anti-SEMA4D) in Combination with Ipilimumab or Nivolumab for Patients with Resectable Pancreatic and Colorectal Cancer. Ann. Surg. Oncol..

[88] Mao J., Yan C., Mei Y., Yao Y., Yang X., Zhuang J., Yu K., Gu G., Zhang H., Zheng Y. (2026). Single-Cell RNA-Seq and Spatial Transcriptomics Characterize CD8^+^ Exhausted T Cells in Pancreatic Ductal Adenocarcinoma. iScience.

[89] Jiang J., Lu Y., Zhang F., Pan T., Zhang Z., Wan Y., Ren X., Zhang R. (2022). Semaphorin 4B Promotes Tumor Progression and Associates with Immune Infiltrates in Lung Adenocarcinoma. BMC Cancer.

[90] Tsai C.-S., Lin H.-Y., Chu P.-Y. (2026). High Semaphorin 4B Expression and Hypomethylation: Drivers of Pancreatic Adenocarcinoma Progression and Immunosuppressive Microenvironment. Formos. J. Surg..

[91] Ling S., Xiong Z., Yan D., Feng J. (2026). PLXNA3 Is Overexpressed and Promotes Cell Proliferation and Migration in Colorectal Cancer. Discov. Oncol..

[92] Zhang Z., Ji S., Zhang B., Liu J., Qin Y., Xu J., Yu X. (2018). Role of Angiogenesis in Pancreatic Cancer Biology and Therapy. Biomed. Pharmacother..

[93] Lucia S.E., Hong S., Abdi R., Kim P. (2026). Heterogeneous Evolution of Pancreatic Cancer Microvasculature Revealed by Longitudinal Intravital Imaging. Cancer Cell Int..

[94] Di Maggio F., Arumugam P., Delvecchio F.R., Batista S., Lechertier T., Hodivala-Dilke K., Kocher H.M. (2016). Pancreatic Stellate Cells Regulate Blood Vessel Density in the Stroma of Pancreatic Ductal Adenocarcinoma. Pancreatology.

[95] Tao J., Yang G., Zhou W., Qiu J., Chen G., Luo W., Zhao F., You L., Zheng L., Zhang T. (2021). Targeting Hypoxic Tumor Microenvironment in Pancreatic Cancer. J. Hematol. Oncol..

[96] Hao X., Ren Y., Feng M., Wang Q., Wang Y. (2021). Metabolic Reprogramming Due to Hypoxia in Pancreatic Cancer: Implications for Tumor Formation, Immunity, and More. Biomed. Pharmacother..

[97] Provenzano P.P., Hingorani S.R. (2013). Hyaluronan, Fluid Pressure, and Stromal Resistance in Pancreas Cancer. Br. J. Cancer.

[98] Lu Z., Weniger M., Jiang K., Boeck S., Zhang K., Bazhin A., Miao Y., Werner J., D’Haese J.G. (2018). Therapies Targeting the Tumor Stroma and the VEGF/VEGFR Axis in Pancreatic Ductal Adenocarcinoma: A Systematic Review and Meta-Analysis. Target Oncol..

[99] Craven K.E., Gore J., Korc M. (2016). Overview of Pre-Clinical and Clinical Studies Targeting Angiogenesis in Pancreatic Ductal Adenocarcinoma. Cancer Lett..

[100] Sadanandam A., Sidhu S.S., Wullschleger S., Singh S., Varney M.L., Yang C.-S., Ashour A.E., Batra S.K., Singh R.K. (2012). Secreted Semaphorin 5A Suppressed Pancreatic Tumour Burden but Increased Metastasis and Endothelial Cell Proliferation. Br. J. Cancer.

[101] Sadanandam A., Rosenbaugh E.G., Singh S., Varney M., Singh R.K. (2010). Semaphorin 5A Promotes Angiogenesis by Increasing Endothelial Cell Proliferation, Migration, and Decreasing Apoptosis. Microvasc. Res..

[102] Casazza A., Finisguerra V., Capparuccia L., Camperi A., Swiercz J.M., Rizzolio S., Rolny C., Christensen C., Bertotti A., Sarotto I. (2010). Sema3E-Plexin D1 Signaling Drives Human Cancer Cell Invasiveness and Metastatic Spreading in Mice. J. Clin. Investig..

[103] Christensen C., Ambartsumian N., Gilestro G., Thomsen B., Comoglio P., Tamagnone L., Guldberg P., Lukanidin E. (2005). Proteolytic Processing Converts the Repelling Signal Sema3E into an Inducer of Invasive Growth and Lung Metastasis. Cancer Res..

[104] Sakurai A., Gavard J., Annas-Linhares Y., Basile J.R., Amornphimoltham P., Palmby T.R., Yagi H., Zhang F., Randazzo P.A., Li X. (2010). Semaphorin 3E Initiates Antiangiogenic Signaling through Plexin D1 by Regulating Arf6 and R-Ras. Mol. Cell. Biol..

[105] Sabag A.D., Bode J., Fink D., Kigel B., Kugler W., Neufeld G. (2012). Semaphorin-3D and Semaphorin-3E Inhibit the Development of Tumors from Glioblastoma Cells Implanted in the Cortex of the Brain. PLoS ONE.

[106] Sierra J.R., Corso S., Caione L., Cepero V., Conrotto P., Cignetti A., Piacibello W., Kumanogoh A., Kikutani H., Comoglio P.M. (2008). Tumor Angiogenesis and Progression Are Enhanced by Sema4D Produced by Tumor-Associated Macrophages. J. Exp. Med..

[107] Fang X., Wang F., Li H., Xiao P., Wang M., Wu N., Yao X., Shi Y. (2026). Revisiting Tumor Vasculature in the Era of Cancer Immunotherapy: From Aberrant Hallmark to Therapeutic Target. Cancer Lett..

[108] Tian L., Goldstein A., Wang H., Ching Lo H., Sun Kim I., Welte T., Sheng K., Dobrolecki L.E., Zhang X., Putluri N. (2017). Mutual Regulation of Tumour Vessel Normalization and Immunostimulatory Reprogramming. Nature.

[109] Martin J.D., Seano G., Jain R.K. (2019). Normalizing Function of Tumor Vessels: Progress, Opportunities, and Challenges. Annu. Rev. Physiol..

[110] Crispim J.G., Silva C.N.P., de Melo Rêgo M.J.B., da Rosa M.M. (2026). Interactions between the Nervous System and PDAC Progression: Regulatory Mechanisms and Therapeutic Implications. Eur. J. Pharmacol..

[111] Lovecek M., Dirimtekin E., Garajová I., Gasparini G., Crippa S., Giovannetti E., Sochorova D., Reyes C.M., Demir I.E., Uysal-Onganer P. (2025). Perineural Invasion in Pancreatic Ductal Adenocarcinoma: Recapitulating Its Importance and Defining Future Directions. United Eur. Gastroenterol. J..

[112] Takahashi R., Ijichi H., Fujishiro M. (2022). The Role of Neural Signaling in the Pancreatic Cancer Microenvironment. Cancers.

[113] Zheng L., Foley K., Huang L., Leubner A., Mo G., Olino K., Edil B.H., Mizuma M., Sharma R., Le D.T. (2011). Tyrosine 23 Phosphorylation-Dependent Cell-Surface Localization of Annexin A2 Is Required for Invasion and Metastases of Pancreatic Cancer. PLoS ONE.

[114] Chen P., Zuo H., Xiong H., Kolar M.J., Chu Q., Saghatelian A., Siegwart D.J., Wan Y. (2017). Gpr132 Sensing of Lactate Mediates Tumor-Macrophage Interplay to Promote Breast Cancer Metastasis. Proc. Natl. Acad. Sci. USA.

[115] Târtea E.-A., Petrescu M., Udriștoiu I., Gheorman V., Biciușcă V., Petrescu A.-R., Ciurea A.-M., Vere C.C. (2023). Clinical Outcomes Depending on Sympathetic Innervation in Pancreatic Cancer. Cancers.

[116] Kobayashi H., Ochiai Y., Arai J., Hata M., Wu F., Sunagawa M., Iida T., Baba T., Malagola E., Tanaka T. (2026). Netrin-1 Promotes Pancreatic Tumorigenesis and Innervation through NEO1. Cancer Res..

[117] Dumartin L., Quemener C., Laklai H., Herbert J., Bicknell R., Bousquet C., Pyronnet S., Castronovo V., Schilling M.K., Bikfalvi A. (2010). Netrin-1 Mediates Early Events in Pancreatic Adenocarcinoma Progression, Acting on Tumor and Endothelial Cells. Gastroenterology.

[118] Haidar H., Bellon A., Sleiman K., Hocine M., Rama N., Gadot N., Carpizo D.R., Mehlen P., Mann F. (2025). Neural Function of Netrin-1 in Precancerous Lesions of the Pancreas. Nat. Commun..

[119] Dudgeon C., Casabianca A., Harris C., Ogier C., Bellina M., Fiore S., Bernet A., Ducarouge B., Goldschneider D., Su X. (2023). Netrin-1 Feedforward Mechanism Promotes Pancreatic Cancer Liver Metastasis via Hepatic Stellate Cell Activation, Retinoid, and ELF3 Signaling. Cell Rep..

[120] Roth G., Artru P., Bouche O., Williet N., Ghelfi J., Turpin A., Lievre A., Blanc J.-F., Evrard C., Bachet J.-B. (2026). Netrin1 Blockade Alleviates Resistance to Chemotherapy in Pancreatic Cancer. Nature.

[121] Francescone R., Barbosa Vendramini-Costa D., Franco-Barraza J., Wagner J., Muir A., Lau A.N., Gabitova L., Pazina T., Gupta S., Luong T. (2021). Netrin G1 Promotes Pancreatic Tumorigenesis through Cancer-Associated Fibroblast-Driven Nutritional Support and Immunosuppression. Cancer Discov..

[122] Biankin A.V., Waddell N., Kassahn K.S., Gingras M.-C., Muthuswamy L.B., Johns A.L., Miller D.K., Wilson P.J., Patch A.-M., Wu J. (2012). Pancreatic Cancer Genomes Reveal Aberrations in Axon Guidance Pathway Genes. Nature.

[123] Nones K., Waddell N., Song S., Patch A.-M., Miller D., Johns A., Wu J., Kassahn K.S., Wood D., Bailey P. (2014). Genome-Wide DNA Methylation Patterns in Pancreatic Ductal Adenocarcinoma Reveal Epigenetic Deregulation of SLIT-ROBO, ITGA2 and MET Signaling. Int. J. Cancer.

[124] Ding C., Li Y., Xing C., Zhang H., Wang S., Dai M. (2020). Research Progress on Slit/Robo Pathway in Pancreatic Cancer: Emerging and Promising. J. Oncol..

[125] Pinho A.V., Van Bulck M., Chantrill L., Arshi M., Sklyarova T., Herrmann D., Vennin C., Gallego-Ortega D., Mawson A., Giry-Laterriere M. (2018). ROBO2 Is a Stroma Suppressor Gene in the Pancreas and Acts via TGF-β Signalling. Nat. Commun..

[126] Göhrig A., Detjen K.M., Hilfenhaus G., Körner J.L., Welzel M., Arsenic R., Schmuck R., Bahra M., Wu J.Y., Wiedenmann B. (2014). Axon Guidance Factor SLIT2 Inhibits Neural Invasion and Metastasis in Pancreatic Cancer. Cancer Res..

[127] Secq V., Leca J., Bressy C., Guillaumond F., Skrobuk P., Nigri J., Lac S., Lavaut M.-N., Bui T.-T., Thakur A.K. (2015). Stromal SLIT2 Impacts on Pancreatic Cancer-Associated Neural Remodeling. Cell Death Dis..

[128] Jin X., Guan X., Mu Y., Wang M., Wang C. (2026). Construction of a SEMA3 Family-Based Model to Predict Prognosis and Molecular Subtypes in Pancreatic Ductal Adenocarcinoma. Discov. Oncol..

[129] Wang L., Wang L., Wang S., Zhou Z., Liu Z., Xu P., Luo X., Wu T., Luo F., Yan J. (2021). N2E4, a Monoclonal Antibody Targeting Neuropilin-2, Inhibits Tumor Growth and Metastasis in Pancreatic Ductal Adenocarcinoma via Suppressing FAK/Erk/HIF-1α Signaling. Front. Oncol..

[130] Casazza A., Kigel B., Maione F., Capparuccia L., Kessler O., Giraudo E., Mazzone M., Neufeld G., Tamagnone L. (2012). Tumour Growth Inhibition and Anti-Metastatic Activity of a Mutated Furin-Resistant Semaphorin 3E Isoform. EMBO Mol. Med..

[131] Patnaik A., Weiss G.J., Leonard J.E., Rasco D.W., Sachdev J.C., Fisher T.L., Winter L.A., Reilly C., Parker R.B., Mutz D. (2016). Safety, Pharmacokinetics, and Pharmacodynamics of a Humanized Anti-Semaphorin 4D Antibody, in a First-In-Human Study of Patients with Advanced Solid Tumors. Clin. Cancer Res..

[132] Shafique M.R., Fisher T.L., Evans E.E., Leonard J.E., Pastore D.R.E., Mallow C.L., Smith E., Mishra V., Schröder A., Chin K.M. (2021). A Phase Ib/II Study of Pepinemab in Combination with Avelumab in Advanced Non-Small Cell Lung Cancer. Clin. Cancer Res..

[133] Mastrantonio R., You H., Tamagnone L. (2021). Semaphorins as Emerging Clinical Biomarkers and Therapeutic Targets in Cancer. Theranostics.

[134] Neufeld G., Mumblat Y., Smolkin T., Toledano S., Nir-Zvi I., Ziv K., Kessler O. (2016). The Role of the Semaphorins in Cancer. Cell Adh. Migr..

[135] Quek C., Pratapa A., Bai X., Al-Eryani G., Pires Da Silva I., Mayer A., Bartonicek N., Harvey K., Maher N.G., Conway J.W. (2024). Single-Cell Spatial Multiomics Reveals Tumor Microenvironment Vulnerabilities in Cancer Resistance to Immunotherapy. Cell Rep..

[136] Miao B., Lih T.-S.M., Hu Y., Zhang H. (2026). Signatures of Pancreatic Ductal Adenocarcinoma Uncovered by Integrative Multi-Omics Analysis. Cancers.

[137] Jin S., Guerrero-Juarez C.F., Zhang L., Chang I., Ramos R., Kuan C.-H., Myung P., Plikus M.V., Nie Q. (2021). Inference and Analysis of Cell-Cell Communication Using CellChat. Nat. Commun..

[138] Efremova M., Vento-Tormo M., Teichmann S.A., Vento-Tormo R. (2020). CellPhoneDB: Inferring Cell-Cell Communication from Combined Expression of Multi-Subunit Ligand-Receptor Complexes. Nat. Protoc..

[139] Fustero-Torre C., Jiménez-Santos M.J., García-Martín S., Carretero-Puche C., García-Jimeno L., Ivanchuk V., Di Domenico T., Gómez-López G., Al-Shahrour F. (2021). Beyondcell: Targeting Cancer Therapeutic Heterogeneity in Single-Cell RNA-Seq Data. Genome Med..

[140] Papadakos S.P., Dedes N., Gkolemi N., Machairas N., Theocharis S. (2023). The EPH/Ephrin System in Pancreatic Ductal Adenocarcinoma (PDAC): From Pathogenesis to Treatment. Int. J. Mol. Sci..

